# Lung scintigraphy in the diagnosis of pulmonary embolism: current methods and interpretation criteria in clinical practice

**DOI:** 10.2478/raon-2013-0060

**Published:** 2014-04-25

**Authors:** Ajda Skarlovnik, Damjana Hrastnik, Jure Fettich, Marko Grmek

**Affiliations:** 1 Department for Vascular Diseases, Ljubljana University Medical Centre, Ljubljana, Slovenia; 2 Department for Nuclear Medicine, Celje General Hospital, Celje, Slovenia; 3 Department for Nuclear Medicine, Ljubljana University Medical Centre, Ljubljana, Slovenia

**Keywords:** pulmonary embolism, lung scintigraphy, interpretation criteria, 0.5 segment mismatch criteria

## Abstract

**Background:**

In current clinical practice lung scintigraphy is mainly used to exclude pulmonary embolism (PE). Modified diagnostic criteria for planar lung scintigraphy are considered, as newer scitigraphic methods, especially single photon emission computed tomography (SPECT) are becoming more popular.

**Patients and methods.:**

Data of 98 outpatients who underwent planar ventilation/perfusion (V/Q) scintigraphy and 49 outpatients who underwent V/Q SPECT from the emergency department (ED) were retrospectively collected. Planar V/Q images were interpreted according to 0.5 segment mismatch criteria and revised PIOPED II criteria and perfusion scans according to PISA-PED criteria. V/Q SPECT images were interpreted according to the criteria suggested in EANM guidelines. Final diagnosis of PE was based on the clinical decision of an attending physician and evaluation of a 12 months follow-up period.

**Results:**

Using 0.5 segment mismatch criteria and revised PIOPED II, planar V/Q scans were diagnostic in 93% and 84% of cases, respectively. Among the diagnostic planar scans readings specificity for 0.5 segment mismatch criteria was 98%, and 99% for revised PIOPED II criteria. V/Q SPECT showed a sensitivity of 100% and a specificity of 98%, without any non-diagnostic cases. In patients with low pretest probability for PE, planar V/Q scans assessed by 0.5 segment mismatch criteria were diagnostic in 92%, and in 85% using revised PIOPED II criteria, while perfusion scintigraphy without ventilation scans was diagnostic in 80%.

**Conclusions:**

Lung scintigraphy yielded diagnostically definitive results and is reliable in ruling out PE in patients from ED. V/Q SPECT has excellent specificity and sensitivity without any non-diagnostic results. Percentage of non-diagnostic results in planar lung scintigraphy is considerably smaller when 0.5 segment mismatch criteria instead of revised PIOPED II criteria are used. Diagnostic value of perfusion scintigraphy according to PISA-PED criteria is inferior to combined V/Q scintigraphy; the difference is evident especially in patients with low pretest probability for PE.

## Introduction

Pulmonary embolism (PE) remains a diagnostic challenge. With the development of modern diagnostic methods, the role of lung scintigraphy in the work up of patients with suspected PE has also changed. In current clinical practice lung scintigraphy is mainly used to exclude PE.^1^,^2^ Recently developments in scintigraphic methods have been made, as well as modified criteria for the interpretation of scans.

For many years, chest radiographs and ventilation/perfusion (V/Q) scintigraphy have been the primary imaging modalities used in the evaluation of patients with suspected acute PE. The revised PIOPED criteria for V/Q scintigraphy currently in use have a reported sensitivity of 41% and a specificity of 97%.^3^ A major problem in clinical practice is the large percentage of scans falling in the category of intermediate (indeterminate) probability of PE.^3^,^4^

Advances in computed tomographic pulmonary angiography (CTPA) have enabled the direct visualization of PE. This technique has emerged as an important diagnostic tool in the evaluation of patients with suspected PE, almost completely replacing scintigraphy in clinical practice in some hospitals.^5^–^8^ However, the suitability of CTPA as a primary diagnostic modality is questionable primarly because of the radiation exposure, certain contraindications and significant percentage of false positive results. In a group of patients with low pretest probability of PE, CTPA gave false positives in as many as 42% of cases.^9^

In 1995 the PISA–PED (Prospective Investigative Study of Acute Pulmonary Embolism Diagnosis) study re-evaluated the role of perfusion scintigraphy alone. According to PISA-PED criteria the perfusion scans were classified into normal, abnormal compatible with PE and abnormal not compatible with PE.^10^ A sensitivity of 92% and a specificity of 87% was reported. In retrospective analysis of data from PIOPED II, perfusion scintigraphy assessed according to PISA-PED criteria, and combined with chest radiography had a sensitivity of 80% and specificity of 96%, none of the study were non-diagnostic.^11^

In 2007 Howarth *et al.* suggested that a more than 0.5 segment V/Q mismatch is sufficient for diagnosis of PE and that such a simplified approach could also reduce the percentage of non-diagnostic scans.^12^

The 2009 European Association for Nuclear Medicine (EANM) guidelines for V/Q lung scintigraphy strongly support the use of Single Photon Emission Computed Tomography (SPECT) V/Q scintigraphy.^13^,^14^ Studies have shown that SPECT has a greater sensitivity and specificity, and a lower number of inconclusive results in the detection of pulmonary embolism compared to planar scans. However, there are several challenges that must be overcome for V/Q SPECT to be successful, including shortening of the acquisition time and a different approach in image reporting.^15^,^16^

The objective of this study was to assess the diagnostic value of lung scintigraphy in outpatients with suspected acute PE using 0.5 segment V/Q mismatch criteria, revised PIOPED II criteria, PISA-PED criteria and V/Q SPECT.

Our aim was: 1. to evaluate the role of V/Q SPECT in the diagnostic algorithm of acute PE; 2. to assess the use of new simplified criteria based on >0.5 segment V/Q mismatch and 3. to assess the value of planar perfusion lung scintigraphy (without ventilation scans) in excluding PE, especially in patients with low pretest probability.

## Patients and methods

The study was retrospective and approved by the National Medical Ethics Committee.

### Patients

Two groups of patients were included in this study. The first group consisted of 98 randomly selected outpatients who were presented in 2010 to the Internistic Emergency Department (IPP) of University Medical Centre in Ljubljana with suspicion of acute PE. In all of the patients a planar V/Q scintigraphy was performed at the Department for Nuclear Medicine in Ljubljana. The second group consisted of 49 randomly selected outpatients who presented in 2010 to IPP of the General Hospital in Celje with suspicion of acute PE and had a V/Q SPECT performed in the Department for Nuclear Medicine in Celje.

Patients with technically inadequate scans, younger than 18 years, on anticoagulant therapy, pregnant women and patients who could not be followed up for 12 months were not included in the study.

Pretest probability was assessed according to Wells’ criteria. D-dimer values were obtained when possible. All patients also had a chest X-ray at the time of presentation.

### Lung scintigraphy

#### Planar ventilation study

Technegas was used for ventilation studies. Images were acquired in the sitting position if possible, in at least four standard projections: posterior, anterior, left and right posterior oblique. The camera was equipped with a low-energy high-resolution (LEHR) collimator. A 256 × 256 pixel matrix was used. The predefined total image acquisition time was 90 seconds, in rare cases it was prolonged (up to 180 seconds).

#### Planar perfusion study

99mTc - Macro Aggregated Albumin (99mTc-MAA) in activity 120–200 MBq was administered intravenously to patients in the supine position. Images were acquired in the sitting position if possible, in at least four standard projections: posterior, anterior, left and right posterior oblique. The camera was equipped with a LEHR collimator. A 256 × 256 pixel matrix was selected for accumulation of at least 600 000 counts per image.

#### V/Q SPECT

Inhalation of Technegas (approximately 20 – 40 MBq accumulated in the lung) and injection of 99mTc-MAA (activity 100–125 MBq) were administered to patient in the supine position. A dual head gamma camera was used, with a total acquisition time of 20 min. 128 projections (64/head) were acquired. The camera was equipped with a LEHR collimator and a 64 × 64 pixel matrix was used.

#### Interpretation criteria

**FIGURE 1.**
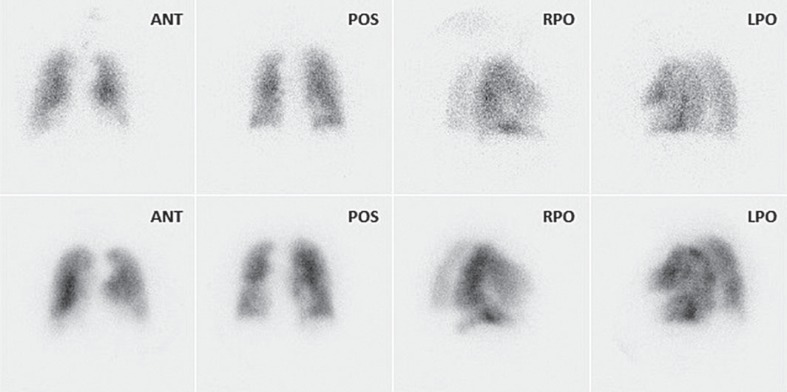
Planar perfusion scintigrams (lower row) with abnormalities, partly matched with abnormalities on planar ventilation scintigrams (upper row) are presented in the picture. The case was assessed as PE negative according to 0,5 segment mismatch criteria, and as non-diagnostic when PIOPED criteria were used. Using PISA-PED criteria the study was assessed as PE positive. (ANT = anterior, POS = posterior, RPO = right posterior oblique, LPO = left posterior oblique). After 12 month follow up the case was closed as PE negative.

Planar lung scans were interpreted independently by 2 qualified nuclear medicine physicians. First, the perfusion scans were interpreted according to PISA-PED criteria with the chest X-ray available but without knowledge of the ventilation data.^10^ This was followed by addition of ventilation data analysis using revised PIOPED criteria.^4^ Separately, V/Q scans were then again interpreted according to 0.5 segment mismatch criteria (Table 1, Figure 1).

V/Q SPECT images were interpreted strictly according to the criteria from EANM guidelines.^13^,^14^ The total extent of perfusion abnormalities compatible with PE was calculated and reported in per cent.

### Final diagnosis

Patient’s final diagnosis was a composite diagnosis based on the clinical decision of the attending physician and evaluation of the 12 months follow-up period when results of all performed investigations (for example CTPA) were taken in consideration.

## Results

### Pretest probability and PE prevalence estimated by final diagnosis

In the first group of 98 outpatients (median age 71 years) who underwent planar V/Q scintigraphy, 4 patients (4%) had high, 32 patients (33%) moderate and 62 patients (63%) low pretest probability for PE. 8 patients (8%) had a final diagnosis of acute PE. 7 patients were given anticoagulant therapy by their attending physician and 1 patient was diagnosed with deep venous thrombosis and PE (on CTPA) in the next two weeks, and was subsequently given anticoagulant therapy. 90 patients (92%) did not have a final diagnosis of acute PE and did not receive anticoagulant therapy within the follow-up period.

In the second group of 49 patients (median age 72 years) who underwent V/Q SPECT, 2 patients (4%) had high, 14 patients (29%) moderate and 33 patients (67%) low pretest probability. 9 patients (18%) had a final diagnosis of acute PE and were given anticoagulant therapy. 40 patients (82%) did not have a final diagnosis of acute PE and did not receive anticoagulant therapy within the follow-up period.

### Lung scintigraphy

The results of scans readings using 0.5 segment mismatch criteria, revised PIOPED II criteria, PISA-PED criteria or V/Q SPECT are presented in Table 2. When assessing planar V/Q scans according to 0.5 segment mismatch criteria, 84 scans (86%) were read as PE negative, 7 scans (7%) were read as PE positive and 7 scans (7%) were non-diagnostic. When assessing planar V/Q scans according to PIOPED criteria, 78 scans (80%) were read as PE negative, 4 scans (4%) were read as PE positive and 16 scans (16%) were non-diagnostic. When assessing planar perfusion scans according to PISA-PED criteria, 68 scans (69%) were read as PE negative, 9 scans (9%) were read as PE positive and 21 scans (22%) were non-diagnostic. When assessing V/Q SPECT scans according to EANM guidelines, 39 scans (80%) were read as PE negative, 10 scans (20%) were read as PE positive and none of the investigation was non-diagnostic.

### Diagnostic value of lung scintigraphy using different interpretative criteria

Positive predictive value (PPV) and negative predictive value (NPV) for lung scintigraphy are presented in Table 3. When assessing planar V/Q scans according to 0.5 segment mismatch criteria, 91 patients (93%) had diagnostic (PE positive or PE negative) result, the PPV was 71% (5/7), while the NPV was 99% (83/84). If only diagnostic scans readings were taken into consideration the sensitivity was 83% and the specificity 98%. When assessing planar V/Q scans according to revised PIOPED II criteria, 82 patients (84%) had diagnostic result, the PPV was 75% (3/4), while the NPV was 99% (77/78). If only diagnostic scans readings were taken into consideration the sensitivity was 75% and the specificity 99%. When assessing planar perfusion scans according to PISA-PED criteria, 77 patients (78%) had diagnostic result, the PPV was 56% (5/9), while the NPV was 99% (67/71). If only diagnostic scans readings were taken into consideration the sensitivity was 83% and the specificity 94%. When using V/Q SPECT, all patients had diagnostic (PE positive or PE negative) result. The sensitivity was 100%, specificity 98%, PPV 90% and NPV 100%.

### Sensitivity of PE positive scan readings and specificity of PE negative scan readings in patients with low pretest probability for PE

In the subgroup of 62 patients with low pretest probability for PE only two patients had PE. Planar scans assessed according to 0.5 segment mismatch, revised PIOPED II and PISA-PED criteria gave definitive readings in 57 patients (92%), 53 patients (85%) and 49 patients (80%), respectively (Table 4). Among patients with definitive scan readings, the sensitivity of PE positive scan findings was 100% with all reading criteria, but PPV for PISA-PED criteria was only 33% (2/6). The specificity of PE negative scan findings was 100%, 100% and 91% (43/47), respectively.

## Discussion

Our study population consisted of outpatients with a predominantly low pretest probability for PE. Therefore it is not surprising that the prevalence of PE in the group of 98 patients who underwent planar scintigraphy was low (9%) and was comparable to that in the population studied by Howarth (13%).^12^ In the group of 49 patients who underwent V/Q SPECT the prevalence of PE was similar to that in the PIOPED II study (19%).^17^

Two thirds of patients had a low pretest probability and 4% had a high pretest probability according to Wells’ criteria, while in PIOPED II study 56% of the patients included had low and 6% had high pretest probability.^18^

### Diagnostic value of planar V/Q scintigraphy

We found out that specificity and NPV of planar V/Q scintigraphy using 0.5 segment mismatch or revised PIOPED II criteria were very good. The results were comparable to the results of PIOPED II and other planar V/Q studies.^3^,^17^,^19^,^20^,^21^ In our study only one patient with PE negative scan reading according to 0.5 segment mismatch criteria and revised PIOPED criteria was later diagnosed with PE. This patient had a high pretest probability – discrepancy between the clinical probability and imaging results was present.

The percentage of non-diagnostic V/Q planar studies in our study (assessed by revised PIOPED criteria) was considerably lower than in the PIOPED II study (16% *vs*. 26.5%), where the studied population was already heavily weighted towards outpatients. When we used 0.5 segment mismatch criteria, the number of non-diagnostic scans was further reduced and did not exceed 8%. This represents a significant improvement in comparison to PIOPED criteria (16% of non-diagnostic scans).

### V/Q SPECT

In our study, V/Q SPECT showed excellent sensitivity, specificity and NPV, comparable to results from other studies, where sensitivities ranged from 80% – 100% and specificities from 93% – 100%.^22^,^23^ V/Q SPECT enables better detection of perfusion defects on the subsegmental level, especially in medial parts of lung. Several studies show better sensitivity and significantly less non-diagnostic results with SPECT than with planar scintigraphy but similar specificity.^14^,^22^,^23^,^24^

Total acquisition times for V/Q SPECT have now decreased to 20–30 minutes for dual head cameras and to 14–20 min for triple head cameras.^25^,^26^ A further option in the workup for acute PE could be use of perfusion SPECT imaging without ventilation. In certain subgroups of patients, *i.e.* in patients with a low pretest probability and normal chest radiographs or pregnant women, ventilation scintigraphy could be performed on following day when needed.

### Perfusion scintigraphy without ventilation

Our results of planar perfusion lung scans readings by PISA-PED criteria were non-diagnostic in a considerably larger percentage compared to V/Q scintigraphy interpreted either by PIOPED or 0.5 segment mismatch criteria (22% *vs.* 16% and 7%, respectively). The percentage of non-diagnostic results was also larger than in the PISA-PED study and retrospective analysis of perfusion scans from PIOPED II study.^17^

### Lung scintigraphy in patients with low pretest probability for PE

Using 0.5 segment mismatch criteria for V/Q scan interpretation 8% of cases were non-diagnostic, and 15% if revised PIOPED II criteria were used. So we recommend the use of 0.5 segment mismatch criteria for scans readings in patients with low pretest probability PE. By using perfusion scintigraphy according to PISA-PED, 20% of investigations were non-diagnostic. There were also 4 false positive results when only lung perfusion scintigraphy was used and none using V/Q scintigraphy. Therefore, our results do not support routine use of only perfusion scintigraphy in patients with low pretest clinical probability. Results based on only perfusion scintigraphy could possibly be improved by gaining more experience with the PISA-PED methodology. Recently, a Chinese multicenter study including 544 patients was published^27^, in which perfusion scintigraphy assessed according to PISA PED criteria showed no non-diagnostic results, with a sensitivity of 86% and a specificity of 81%.

### Lung scintigraphy and the diagnostic algorithm for acute PE in outpatients

In all patients with suspected acute PE, assessment of pretest probability according to Wells’ criteria is strongly recommended.^28^ Depending on clinical probability, especially D-dimer, further diagnostic imaging is indicated. Echocardiography and Duplex examination of lower extremity veins are now standard and easily accessible in an ED clinical setting.

According to our results, planar V/Q scintigraphy can reliably rule out PE. Its results are not inferior to CTA^9^ and could be recommended as the imaging test of choice first of all in patients with a low clinical probability. Use of planar perfusion scintigraphy alone might be considered in patients who cannot ventilate adequately and those with normal findings on plain chest radiograph.^29^,^30^ Excellent diagnostic accuracy of V/Q SPECT and reduction of the acquisition time make this method clinically useful for diagnosis of acute PE in an ED setting also in patients with higher clinical probability.^31^

Additional workup is necessary when the clinical probability is inconsistent with the imaging results regardless of used modality.

## Conclusions

In the outpatient population of an emergency department PE can be reliably ruled out using planar V/Q lung scintigraphy or V/Q SPECT.

V/Q SPECT has excellent sensitivity and specificity without non-diagnostic results.

Percentage of non-diagnostic results in planar lung scintigraphy is considerably smaller when 0.5 segment mismatch criteria instead of revised PIOPED II criteria are used.

Diagnostic value of perfusion scintigraphy according to PISA-PED criteria is inferior to combined V/Q scintigraphy; the difference is evident especially in patients with low pretest probability for PE.

## Figures and Tables

**TABLE 1.** Criteria used for planar lung scans interpretation in patients with suspicion of acute pulmonary embolism: 0.5 segment mismatch criteria, revised PIOPED II criteria and PISA-PED criteria. (* prominent hilum, cardiomegaly, elevated diaphragm, linear atelectasis or costophrenic angle effusion)

	**0.5 segment mismatch criteria**	**Revised PIOPED II criteria**	**PISA-PED criteria**
**PE positive**	≥2 segments of V/Q mismatch ≥3 V/Q mismatch defects >50% of segment	≥2 segments of V/Q mismatch	≥1 wedge-shaped Q defect(s) corresponding to anatomic regions of the lung
**PE negative**	Nonsegmental perfusion abnormalities^*^ Q defect smaller than corresponding radiographic lesion 1 V/Q mismatch defect ≤50% of segment Stripe sign	Nonsegmental perfusion abnormalities* Q defect smaller than corresponding radiographic lesion ≥2 matched V/Q defects with regionally normal chest regionally normal chest radiograph 1–3 small segmental perfusion defects (<25% of segment) Solitary triple matched defect in the mid or upper lung zone confined to a single segment Stripe sign Large pleural effusion	Other than wedge shaped Q defects Presence of impressions caused by enlarged heart, hila or mediastinum on an otherwise normal scan No Q defects
**Nondiagnostic**	All other findings	All other findings	All other findings

PE = pulmonary embolism; V/Q = ventilation / perfusion

**TABLE 2.** Number and percentage of PE negative, non - diagnostic and PE positive readings when lung scans were interpreted using 0.5 segment mismatch criteria, revised PIOPED II criteria or PISA-PED criteria and V/Q SPECT

**Reading criteria (method)**	**N**	**PE negative readings**	**Non-diagnostic readings**	**PE positive readings**
**0.5 segment mismatch (planar V/Q scintigraphy)**	98	84 (86%)	7 (7%)	7 (7%)
**Revised PIOPED II (planar V/Q scintigraphy)**	98	78 (80%)	16 (16%)	4 (4%)
**PISA-PED (planar Q scintigraphy)**	98	68 (69%)	21 (22%)	9 (9%)
**V/Q SPECT**	49	39 (80%)	0 (0%)	10 (20%)

PE = pulmonary embolism; V/Q = ventilation / perfusion

**TABLE 3.** Positive predictive value (PPV) and negative predictive value (NPV) for 0.5 segment mismatch criteria, revised PIOPED II criteria, PISA-PED criteria and V/Q SPECT in lung scans interpretations are presented in the table

**Reading criteria (method)**	**Diagnostic readings**	**PPV**	**NPV**
**0.5 segment mismatch (planar V/Q scintigraphy)**	93% (91/98)	71% (5/7)	99% (83/84)
**Revised PIOPED II (planar V/Q scintigraphy)**	84% (82/98)	75% (3/4)	99% (77/78)
**PISA-PED (planar Q scintigraphy)**	78% (77/98)	56% (5/9)	99% (67/68)
**V/Q SPECT**	49/49 (100%)	90% (9/10)	100% (39/39)

PE = pulmonary embolism; V/Q = ventilation / perfusion

**TABLE 4.** Number and percentage of PE negative, non- diagnostic and PE positive studies in patients with low pretest probability for PE when lung scans were interpreted according to 0.5 segment mismatch criteria, revised PIOPED II criteria or PISA-PED criteria

**Reading criteria (method)**	**N**	**PE negative readings**	**Non-diagnostic readings**	**PE positive readings**
**0.5 segment mismatch (planar V/Q scintigraphy)**	62	55 (89%)	5 (8%)	2 (3%)
**Revised PIOPED II (planar V/Q scintigraphy)**	62	51 (82%)	9 (15%)	2 (3%)
**PISA-PED (planar Q scintigraphy)**	62	43 (70%)	13 (20%)	6 (10%)

PE = pulmonary embolism; V/Q = ventilation / perfusion

## References

[1] British Thoracic Society Standards of Care Committee Pulmonary Embolism Guideline Development Group (2003). British Thoracic Society guidelines for the management of suspected acute PE. Thorax.

[2] Kluetz PG, White CS (2006). Acute Pulmonary embolism: imaging in the emergency department. Rad Clin N Am.

[3] The PIOPED Investigators (1990). Value of the ventilation/perfusion scan in acute pulmonary embolism. Results of the prospective investigation of pulmonary embolism diagnosis (PIOPED). JAMA.

[4] Stein PD, Woodard PK, Weg JG, Wakefield TW, Tapson VF, Sostman HD (2006). PIOPED II investigators. Diagnostic pathways in acute pulmonary embolism: reccomendations of the PIOPED II investigators. Am J Med.

[5] Stein PD, Sostman HD, Bounameaux H, Buller HR, Chenevert TL, Dalen JE (2008). Challenges in the diagnosis acute pulmonary embolism. Am J Med.

[6] Musset D, Parent F, Meyer G, Maitre S, Girard P, Leroyer C (2002). Diagnostic strategy for patients with suspected pulmonary embolism: a prospective multicentre outcome study. The Lancet.

[7] Van Rossum AB, Pattynama PM, Ton ER, Treurniet FE, Arndt JW, van Eck B (1996). Pulmonary embolism: validation of spiral CT angiography in 149 patients. Radiology.

[8] Bešlić Š, Dalagija F, Đurović V (2005). Multislice computed tomography of pulmonary embolism: spectrum of findings. Radiol Oncol.

[9] Stein PD, Fowler SE, Goodman LR, Gottschalk A, Hales CA, Hull RD (2006). Multidetector computed tomography for acute pulmonary embolism. N Engl J Med.

[10] Miniati M, Pistolesi M, Marini C, Di Ricco G, Formichi B, Prediletto R (1996). Value of perfusion lung scan in the diagnosis of pulmonary embolism: results of the prospective investigative study of acute pulmonary embolism diagnosis (PISA-PED). Am J Respir Crit Care Med.

[11] Miniati M, Sostman HD, Gottschalk A, Monti S, Pistolesi M (2008). Perfusion lung scintigraphy for the diagnosis of pulmonary embolism: a reappraisal and review of the prospective investigative study of acute pulmonary embolism diagnosis methods. Sem Nucl Med.

[12] Howarth DM, Booker JA, Voutnis DD (2006). Diagnosis of pulmonary embolus using ventilation/perfusion lung scintigraphy: more than 0,5 segment of ventilation/perfusion V/Q mismatch is sufficient. Intern Med J.

[13] Bajc M, Neilly JB, Miniati M, Schuemichen C, Meignan M, Jonson B (2009). EANM guidelines for ventilation/perfusion scintigraphy Part 1. Pulmonary imaging with ventilation/perfusion single photon emission tomography. Eur J Nucl Med Mol Imaging.

[14] Bajc M, Neilly JB, Miniati M, Schuemichen C, Meignan M, Jonson B (2009). EANM guidelines for ventilation/perfusion scintigraphy Part 2. Algorithms and clinical considerations for diagnosis of pulmonary emboli with V/P SPECT and MDCT. Eur J Nucl Med Mol Imaging.

[15] Reinartz P, Wildberger JE, Schaefer W, Nowak B, Mahnken AH, Buell U (2004). Tomographic imaging in the diagnosis of pulmonary embolism: a comparison between V/Q lung scintigraphy in SPECT technique and multislice spiral CT. J Nucl Med.

[16] Jögi J, Jonson B, Ekberg M, Bajc M (2010). V/P SPECT with 99Tc-DTPA versus Technegas: a head-to-head study in obstructive and nonobstructive disease. J Nucl Med.

[17] Sostman HD, Miniati M, Gottschalk A, Matta F, Stein PD, Pistolesi M (2008). Sensitivity and specificity of perfusion scintigraphy combined with chest radiography for acute pulmonary embolism in PIOPED II. J Nucl Med.

[18] Stein PD, Beemath A, Matta F, Weg JG, Yusen RD, Hales CA (2007). Clinical characteristics of patients with acute pulmonary embolism: data from PIOPED II. Am J Med.

[19] Sostman HD, Stein PD, Gottschalk A, Matta F, Hull R, Goodman L (2008). Acute pulmonary embolism: sensitivity and specificity of ventilation-perfusion scintigraphy in PIOPED II study. Radiology.

[20] Anderson DR, Kahn SR, Rodger MA, Kovacs MJ, Morris T, Hirsch A (2007). Computed tomographic pulmonary angiography vs ventilation–perfusion lung scanning in patients with suspected pulmonary embolism. JAMA.

[21] Stein PD, Henry JW, Gottschalk A (1993). Mismatched vascular defects. An easy alternative to mismatched segmental equivalent defects for the interpretation of ventilation/perfusion lung scans in pulmonary embolism. Chest.

[22] Collart JP, Roelants V, Vanpee D, Lacrosse M, Trigaux JP, Delaunois L (2002). Is a lung perfusion scan obtained by using single photon emission computed tomography able to improve the radionuclide diagnosis of pulmonary embolism?. Nucl Med Commun.

[23] Stein PD, Freeman LM, Sostman HD, Goodman LR, Woodard PK, Naidich DP (2009). SPECT in acute pulmonary embolism. J Nucl Med.

[24] Bajc M (2005). Value of ventilation/perfusion SPECT detecting extensive pulmonary embolism in patient with pneumonia. Thromb Haemost.

[25] Stein PD, Goodman LR, Hull RD, Dalen JE, Matta F (2012). Diagnosis and management of isolated subsegmental pulmonary embolism: review and assessment of the options. Clin App Thromb Haem.

[26] Reinartz P, Schirp U, Zimny M, Sabri O, Nowak B, Schäfer W (2001). Optimizing ventilation-perfusion lung scintigraphy: parting with planar imaging. Nuklearmedizin.

[27] He J, Wang F, Dai HJ, Li M, Wang Q, Yao Z (2012). Chinese multi-center study of lung scintigraphy and CT pulmonary angiography for the diagnosis of pulmonary embolism. Int J Cardiovasc Imaging.

[28] Miniati M, Bottai M, Monti S (2005). Comparison of three clinical models for predicting the probability of pulmonary embolism. Medicine (Baltimore).

[29] Stein PD, Sostman HD, Dalen JE, Bailey DL, Bajc M, Goldhaber SZ (2011). Controversies in diagnosis of pulmonary embolism. Clin App Thromb Haem.

[30] Glaser JE, Chamarthy M, Haramati LB, Esses D, Freeman LM (2011). Successful and safe implementation of a trinary interpretation and reporting strategy for V/Q lung scintigraphy. J Nucl Med.

[31] Sostman HD, Pistolesi M (2011). Scintigraphy for pulmonary embolism: too old to rock’n roll, too young to die?. J Nucl Med.

